# Efficacy of a Metalloproteinase Inhibitor in Spinal Cord Injured Dogs

**DOI:** 10.1371/journal.pone.0096408

**Published:** 2014-05-01

**Authors:** Jonathan M. Levine, Noah D. Cohen, Michael Heller, Virginia R. Fajt, Gwendolyn J. Levine, Sharon C. Kerwin, Alpa A. Trivedi, Thomas M. Fandel, Zena Werb, Augusta Modestino, Linda J. Noble-Haeusslein

**Affiliations:** 1 Department of Small Animal Clinical Sciences, College of Veterinary Medicine and Biomedical Sciences, Texas A&M University, College Station, Texas, United States of America; 2 Department of Large Animal Clinical Sciences, College of Veterinary Medicine and Biomedical Sciences, Texas A&M University, College Station, Texas, United States of America; 3 Department of Bioengineering, University of California San Diego, San Diego, California, United States of America; 4 Department of Veterinary Physiology and Pharmacology, College of Veterinary Medicine and Biomedical Sciences, Texas A&M University, College Station, Texas, United States of America; 5 Department of Veterinary Pathobiology, College of Veterinary Medicine and Biomedical Sciences, Texas A&M University, College Station, Texas, United States of America; 6 Department of Neurological Surgery, University of California San Francisco, San Francisco, California, United States of America; 7 Department of Anatomy, University of California San Francisco, San Francisco, California, United States of America; 8 Department of Physical Therapy and Rehabilitation, University of California San Francisco, San Francisco, California, United States of America; Rutgers-Robert wood Johnson Medical School, United States of America

## Abstract

Matrix metalloproteinase-9 is elevated within the acutely injured murine spinal cord and blockade of this early proteolytic activity with GM6001, a broad-spectrum matrix metalloproteinase inhibitor, results in improved recovery after spinal cord injury. As matrix metalloproteinase-9 is likewise acutely elevated in dogs with naturally occurring spinal cord injuries, we evaluated efficacy of GM6001 solubilized in dimethyl sulfoxide in this second species. Safety and pharmacokinetic studies were conducted in naïve dogs. After confirming safety, subsequent pharmacokinetic analyses demonstrated that a 100 mg/kg subcutaneous dose of GM6001 resulted in plasma concentrations that peaked shortly after administration and were sustained for at least 4 days at levels that produced robust *in vitro* inhibition of matrix metalloproteinase-9. A randomized, blinded, placebo-controlled study was then conducted to assess efficacy of GM6001 given within 48 hours of spinal cord injury. Dogs were enrolled in 3 groups: GM6001 dissolved in dimethyl sulfoxide (n = 35), dimethyl sulfoxide (n = 37), or saline (n = 41). Matrix metalloproteinase activity was increased in the serum of injured dogs and GM6001 reduced this serum protease activity compared to the other two groups. To assess recovery, dogs were *a priori* stratified into a severely injured group and a mild-to-moderate injured group, using a Modified Frankel Scale. The Texas Spinal Cord Injury Score was then used to assess long-term motor/sensory function. In dogs with severe spinal cord injuries, those treated with saline had a mean motor score of 2 (95% CI 0–4.0) that was significantly (P<0.05; generalized linear model) less than the estimated mean motor score for dogs receiving dimethyl sulfoxide (mean, 5; 95% CI 2.0–8.0) or GM6001 (mean, 5; 95% CI 2.0–8.0). As there was no independent effect of GM6001, we attribute improved neurological outcomes to dimethyl sulfoxide, a pleotropic agent that may target diverse secondary pathogenic events that emerge in the acutely injured cord.

## Introduction

Matrix metalloproteinases (MMPs) are endopeptidases that degrade the extracellular matrix [1]. Several members of the MMP family, including MMP-9 (gelatinase B) and MMP-12, have been implicated in early secondary pathogenesis after spinal cord injury (SCI) [2]–[4]. These MMPs are released by local cells as well as by infiltrating leukocytes and result in reduced cell-cell adhesion, disruption of the blood-spinal cord barrier, up-regulation of pro-inflammatory cytokines, and demyelination [1], [4], [5].

Early blockade of MMPs confers neuroprotection after SCI [2], [6], [7]. Short-term administration of the broad-spectrum MMP inhibitor, GM6001, results in sparing of white matter and improves locomotor function when the drug is given over the first 3 days post-injury [2]. Several lines of evidence suggest that one likely target of GM6001 is MMP-9. This protease is not actively expressed in the uninjured spinal cord and is up-regulated over the first 3 days post-injury, corresponding to the time-course for infiltration of neutrophils [8]. While there are local sources of MMP-9, including glia and endothelial cells, neutrophil depletion studies confirm that these leukocytes are the major source of MMP-9 in the acutely injured cord [7]. As this protease is not complexed with tissue inhibitor of MMP-1, degranulation of neutrophils results in release of activated MMP-9 [9], which then may disrupt the barrier and facilitate transmigration of leukocytes into the injured spinal cord. It thus is not surprising that early administration of GM6001 attenuates the trafficking of neutrophils into the injured spinal cord and stabilizes the blood-spinal cord barrier [2]. There are other members of the MMP family that are also determinants of recovery after SCI including MMP-12 and ADAM-8 (a disintegrin and metalloprotease domain) [3]. Thus, broad inhibitors of MMPs may offer greater benefit than specific inhibitors of these proteases.

In this study, we have used dimethyl sulfoxide (DMSO) in combination with GM6001 [10], [11]. While DMSO is commonly used as a vehicle to increase solubility of a drug, it has been reported to have neuroprotective properties in traumatic brain injury and SCI [12], [13]. The putative neuroprotective activity of DMSO is thought to arise from its ability to block voltage-sensitive sodium channels and calcium influx into cells, and mitigate opening of ionotropic channels that are activated by glutamate [14].

Few studies have considered a pre-clinical platform involving dogs with naturally occurring SCIs resulting from intervertebral disk herniation (IVDH) [15]–[17]. This approach mimics pathologic aspects of human SCI including compressive/contusive injuries and a pro-inflammatory response that includes the infiltration of neutrophils and up-regulation of MMP-9 [18]–[20]. Moreover, these naturally-occurring injuries provide a means for studying therapeutics in the challenging context of varying degrees of injury severity, common in human SCI, but without confounding factors such as anesthetics that are necessary during creation of injury in experimental models.

Here we evaluate the efficacy of GM6001 in dogs with IVDH. Based on a double-blind, randomized, placebo-controlled trial, consisting of 3 groups (GM6001 in DMSO, DMSO alone, or saline) we show enhanced neurological recovery in dogs sustaining severe SCIs when treated acutely with GM6001 solubilized in DMSO or DMSO alone, relative to the saline group. Such findings implicate DMSO in improving neurological recovery, which is consistent with its reported ability to attenuate secondary pathogenic events in various models of neurotrauma [14].

## Materials and Methods

### Study Design and Inclusion Criteria

A preliminary drug tolerance study was constructed based on Food and Drug Administration guidelines (http://www.fda.gov/AnimalVeterinary/default.htm) and performed in 4 healthy, purpose-bred Beagles. Ten healthy, purpose-bred Beagles were obtained to evaluate pharmacokinetics (PK); this sample size was based on similar animal studies and general recommendations for canine PK investigations [21].

Guidelines for the conduct of SCI trials developed by the International Campaign for Cures of Spinal Cord Injury Paralysis were utilized to assist with the design of a randomized, double-blinded (clinicians and clients were unaware of treatment group), placebo-controlled canine trial including inclusion/exclusion criteria, randomization protocol, data handling, and the *a priori* definition of outcome metrics and statistical approaches.[22]. Consolidated Standards of Reporting Trials (CONSORT) Statement Guidelines were used to assist with trial performance and data reporting [23], [24]. Client-owned dogs with IVDH-associated SCI, admitted to the Texas A&M University Veterinary Medical Teaching Hospital between September 2008 and February 2012, were recruited. The study interval was selected to generate a sample size of >100 dogs, which was considered robust based on previous human phase II and III SCI studies [25], [26], animal model studies of SCI using MMP blockers [2], and completed canine SCI studies [27], [28]. A formal power calculation was not performed due to the absence of a phase I canine study examining the effects of GM6001.

Dogs had to meet the following criteria to be included in the clinical trial population: 1) duration of SCI was required to be ≤48 hours; 2) IVDH-associated SCI had to result in non-ambulatory paraparesis or paraplegia at enrollment; 3) IVDH-associated SCI had to be identified between the T8-L6 vertebral articulations and treated via surgical decompression. The exclusion criteria were: 1) concurrent disseminated neoplasia or systemic inflammation; 2) a history of recent breeding/pregnancy; and, 3) glucocorticoid treatment within 7 days of SCI.

The primary outcome of the clinical trial was a validated ordinal SCI score (the Texas Spinal Cord Injury Score [TSCIS]) conducted at 42 days post-injury [29]. The secondary outcome was TSCIS at 3 days after SCI. Dogs were stratified into those with behaviorally severe SCI (absent pelvic limb movement and deep nociception) and those with mild-to-moderate SCI (intact pelvic limb deep nociception with or without movement) at study entry to examine primary and secondary outcomes. This *a priori* stratification was utilized because a substantially lower proportion of dogs with severe SCI recover independent ambulation at long-term follow-up time-points (approximately 50–60%) in comparison to dogs with mild-to-moderate SCI (approximately 85 to 95%); thus, injury severity might influence the ability to detect treatment-related effects [30]–[33].

### Ethics Statement

All animal procedures were approved by the Texas A&M University Institutional Animal Care and Use Committee (AUP 2007–115; AUP 2011–057; AUP-2011–145) and in the case of client-owned dogs were performed with signed consent. All studies adhered to the National Institutes of Health Guide for the Care and Use of Laboratory Animals.

### Drug Preparation, Drug Tolerance, and Pharmacokinetic Procedures

For all canine studies, GM6001 (SAI Advantium, Hyderabad, India) was dissolved in 90% DMSO (Domoso, Fort Dodge Corp, Fort Dodge, IA) at a concentration of 250 mg/mL. The solution was sterilized using a 25-mm syringe filter with 0.22-µm HT Tuffryn membrane (Pall Corporation, East Hills, NY).

Dogs, participating in the drug tolerance study, were acclimatized for 14 days and then randomized as follows: DMSO (at a volume equivalent to that present in a 100 mg/kg GM6001 treatment), 100 mg/kg GM6001, 150 mg/kg GM6001, or 300 mg/kg GM6001 subcutaneously (SC) every 12 hours for 3 days. The doses of GM6001 were selected to exceed those reported previously in a murine model of SCI [2]. A SC route of administration was selected as 1) GM6001 does not remain solubilized in DMSO when exposed to hydrophilic solutions such as blood, prohibiting intravenous delivery and 2) intraperitoneal drug administration is not generally permitted in client-owned dogs at our institution, due to challenges in managing any local drug reactions. Adverse event monitoring was performed for 7 days following the completion of drug administration. All dogs had physical examinations, injection site evaluations, and assessment of food and water intake twice daily. A complete blood count, serum biochemistry profile, urinalysis, and coagulation profile were performed 3 and 7 days following the completion of drug administration. Following the completion of this study, the vehicle and 300 mg/kg GM6001 dogs were euthanized via intravenous administration of 120 mg/kg pentobarbital (Fatal Plus, Vortech Pharmaceuticals, Dearborn, MI). The brain, heart, liver, kidney, lung, intestine, and injection sites were evaluated.

For PK assessments, a single 100 mg/kg SC administration of GM6001 was delivered in 5 dogs with 5 additional dogs receiving a second 100 mg/kg SC of GM6001, 12 hours following the first dose. In dogs with single dosing, serial plasma samples were obtained at 5, 15, and 30 minutes and 1, 2, 3, 6, 12, 24, 36, 48, and 96 hours after GM6001 delivery. In dogs with multiple dosing, blood samples were collected shortly after the second dose, and then at 24, 48, 72, and 96 hours. All samples were stored in a −80^ο^C freezer until analyzed by high performance chromatography (Thermo Electron Co., Waltham, MA) and tandem mass spectroscopy (MDS-Sciex/Applied Biosystems API3000, Concord, ONT) (LC-MS/MS). Concentrations of GM6001 (m/z 389.0→356.0) were determined, using MMP Inhibitor III (m/z 364.0→356.0, Calbiochem, Billerica, MA) as the internal standard. A standard curve was created with blank dog plasma at concentrations 10.0 to 10,204.0 ng/mL, with linear regression and weighting of concentrations (1/x^2^). After thawing and addition of internal standard (300 µL of 100 ng/mL in 0.5% acetic acid in methanol), plasma samples or standards (100 µL) were centrifuged and reconstituted with 30/70 methanol/10 mM ammonium formate buffer, pH 3.0, for protein precipitation. Supernatant (100 µL) was collected, vortexed, and refrigerated until injection on LC-MS/MS. The mobile phase consisted of 0.1% formic acid in deionized water (A) and acetonitrile/methanol/formic acid (40∶60:0.1, v/v/v) (B) with a flow rate of 0.30 mL/minute using a linear gradient starting at 40% B from 0 to 0.01 minutes, to 80% B at 1.5 minutes, to 90% B at 3.5 minutes, to 40% B at 3.6 minutes, with a total run time of 5 minutes.

### Randomized, Placebo Controlled Study in Dogs with IVDH-associated SCI

Dogs, enrolled in the clinical trial, had physical and neurological examinations, complete blood count and serum biochemistry profile. Anesthesia was induced with propofol (Rapinovet, Schering-Plough Animal Health Corp, Union, NJ) and maintained with inhalant sevoflurane (SevoFlo, Abbott Laboratories, North Chicago, IL). Diagnostic imaging consisting of myelography, computed tomography (CT), or MRI was performed to identify IVDH. Cerebrospinal fluid (CSF) was collected from the cisterna magna for routine analysis and a 200-µL aliquot was stored at −80^ο^C for determination of MMP-2/MMP-9 activity. Six mL of whole blood were obtained at the time of CSF collection and 3 days following treatment delivery; serum was isolated and frozen at −80^ο^C.

Immediately after collection of CSF and blood, dogs were randomized to receive 100 mg/kg GM6001+ DMSO, DMSO, or saline placebo. The dose of both DMSO and saline was 0.4 mL/kg, a volume equivalent to that of 100 mg/kg GM6001+DMSO; this approach was taken to maintain blinding. A randomization sequence was developed prior to the initiation of this trial and randomization was accomplished by blocking the dogs by gender status in a 1∶1∶1 ratio to each of the treatment groups. Sealed envelopes contained treatment allocations and were delivered to a central location where treatments were formulated by individuals not involved in the assessment of animals. Treatments were covered and marked only with animal identifiers to ensure blinding.

Following surgical decompression, all dogs were recovered in an intensive care unit for 24 hours and during that time were provided post-operative opioid analgesia and bladder evacuation. Physical rehabilitation protocols were standardized for dogs participating in this study. Dogs received thoracic limb and pelvic limb passive range of motion exercises beginning 24 hours post-operatively and until dogs could independently ambulate. Each limb was gently flexed and extended at the carpal, elbow, and hip joints in 3 sets of 10 repetitions, 2 times daily. Supported standing exercises were performed twice daily for 5 minutes by placing a sling immediately cranial to the pelvic limbs and continued until dogs could independently ambulate. Dogs that were non-ambulatory were walked using a sling placed immediately cranial to the pelvic limbs for 5 minutes twice daily. Independently ambulatory dogs were permitted to walk on a leash for 5 minutes 3–4 times per day during hospitalization and were allowed to continue this activity until 42-day re-check. Participating dogs were housed in cages that permitted limited additional activity until 42-day re-check evaluation.

### Neurological Assessments

Clinicians responsible for neurologic scoring were blinded to treatment assignments. Two ordinal SCI scores were used to address injury severity at study entry, day 3 post-treatment, and day 42 post-treatment. In both scoring systems, dogs were considered ambulatory if they could spontaneously rise, bear weight, and take at least 10 steps without falling. Dogs that were non-ambulatory had pelvic limb movement evaluated using tail support. Postural responses were evaluated by placing the dorsum of the pes on a non-slick surface while manually supporting the animal and waiting for limb correction. Pelvic limb deep and superficial nociception were evaluated by applying hemostats to a nail-bed or interdigital webbing, respectively and evaluating for the presence of a behavioral or physiological response.

A modified Frankel scale (MFS) was developed to broadly parallel the American Spinal Cord Injury Association Impairment Scale (AIS) [15], [29]. Dogs were scored as paraplegic with absent deep nociception (0; equivalent to AIS A), paraplegic with absent superficial nociception (1; equivalent to AIS B), paraplegic with intact nociception (2; equivalent to AIS B), or non-ambulatory with identifiable pelvic limb movement (3; equivalent to AIS C). The MFS was not a primary trial outcome, but instead was used to describe the baseline population (overall and by treatment group) and to stratify the study population for analysis.

The Texas Spinal Cord Injury Score (TSCIS) was used to assess pelvic limb gait, posture and nociception. This is a more refined scale than the MFS [15] with a larger array of sub-categories, including gait assessment that parallels the Basso, Beattie, Bresnahan Scale [34]. The TSCIS gait score ranges from 0 to 6 in each pelvic limb and correlates to the degree of limb protraction and weight bearing. The gait classifications include: no voluntary movement seen when the dog is supported (score = 0); intact limb protraction with no ground clearance (1); intact limb protraction with inconsistent ground clearance (2); intact protraction with ground clearance >75% of steps (3); ambulatory with consistent ground clearance and significant paresis-ataxia that results in occasional falling (4); ambulatory with consistent ground clearance and mild paresis-ataxia that does not result in falling (5); and normal gait (6). Pelvic limb postural responses using the TSCIS were scored in each limb as absent (0), delayed (1, correction occurred >1 second after positioning), and present (2). Nociception was scored in each limb as absent (0), deep nociception only present (1), or both deep and superficial nociception present (2).

### Magnetic Resonance Imaging (MRI)

Vertebral column MRI was performed on enrolled dogs, except in cases where animals were evaluated outside of normal operating hours or the scanner was unavailable due to mechanical failure. Between September 2008 and July 2011, a 1.0 T system (Siemens Magnetom, Malvern, PA) was utilized to acquire images; for the remainder of the trial images were generated using a 3.0 T MRI (Simens Verio, Malvern, PA). Dogs that received MRI had sagittal T2-weighted (T2W) images reviewed by 1 investigator (JML) using commercially available software (eFILM, Merge Healthcare, Chicago, IL) prior to un-blinding. The acquisition parameters for sagittal T2W images generated at 1T and 3T included a repetition time of 3500 ms, echo time of 90 ms, and slice thickness of 2.0 mm. The presence of spinal cord T2W hyperintensity was determined by visually comparing injured parenchyma to surrounding spinal cord. This technique has been used extensively in human and canine MRI studies and produces repeatable results that correlate with behavioral measures of SCI severity and recovery [35], [36].

### MMP-2/MMP-9 Activity in CSF and Serum

CSF and serum samples (n = 16/treatment group) were randomly selected at the end of the trial by computerized sorting on random numbers. Purpose-bred Beagle dogs (n = 5) were sampled as controls. Serum samples with overt hemolysis were excluded from analysis.

Activity of MMP-2 and MMP-9 in serum and CSF samples was assessed in a blinded manner using a previously developed electrophoretic method [37]–[39] that included a synthetic peptide (AAPPtec, Louisville, KY) (sequence: Ac-NGDPVGLTAGAGK-NH2), tagged with a fluorophore BODIPY-FL-SE (Invitrogen, Carlsbad, CA). The substrate was mixed with either serum or CSF, with phosphate buffered saline as the negative control. After reacting for 1 hour, aliquots were loaded onto 20% polyacrylamide gels and the samples were electrophoresed. Gels were imaged using a BioDoc-It M-26 transilluminator (UVP, Upland, CA, USA). The image was scanned in a Storm 840 workstation (Molecular Dynamics, Sunnyvale, CA, USA) with ImageQuant v5.2 software and fluorescent signal was quantified using ImageJ (1.440, National Institutes of Health, Bethesda, MD).

To assess ability of GM6001 to inhibit MMP-9, activity (described above) was determined using human recombinant MMP-9 (Sigma, St. Louis, MO) that was serially diluted in DMSO to final concentrations of 0.01 µM to 200 µM. Controls consisted of enzyme and substrate only and substrate and GM6001 only.

### Statistical Analyses

Noncompartmental pharmacokinetic analysis was performed (Phoenix WinNonLin 6.3, Pharsight, St. Louis, MO), and estimates of the parameters of T_max_, C_max_, T_1/2_, and area AUC_0-obs_ and AUC_0-∞_ were calculated for the single dose.

Activities of MMP-2/MMP-9 in CSF and serum were compared between healthy control dogs and the dogs with SCI using the Wilcoxon rank-sum test. The Wilcoxon rank-sum test was also used to compare CSF and serum MMP-2/MMP-9 activities between dogs with and without selected characteristics that were potential modifiers of MMP-2/MMP-9 (i.e., age, breed, sex, and markers of disease duration or severity). To compare serum MMP-2/MMP-9 values among treatment groups, the serum MMP-2/MMP-9 activities were converted to ranks, and the ranks were compared using a generalized linear model; multiple pair-wise comparisons between treatments were made using the method of Sidak. Model fit was assessed graphically using diagnostic plots of residuals.

For the clinical trial data, a strategy for analysis of data was developed *a priori*, including our decision to stratify the population based on SCI severity at admission. Baseline characteristics were compared among the 3 treatment groups to determine whether there was any evidence of differences among groups. Categorical variables were compared using chi-squared analysis and continuous or ordinal variables were compared using Kruskal-Wallis tests. The primary outcome for the trial was the TSCIS score on day 42. The TSCIS on day 3 was considered a secondary outcome. The association of TSCIS with treatment group and other individual variables was assessed using generalized linear modeling. Individual variables significantly associated with TSCIS were analyzed using multivariable generalized linear modeling using maximum likelihood estimating methods. Multiple comparisons among groups were adjusted using the method of Sidak. Model fit was assessed graphically using diagnostic plots of residuals. Comparisons of proportions among treatments groups (e.g., frequency of adverse events) were made using chi-squared or, when appropriate, Fisher’s exact tests. Significance was set at P<0.05 for all analyses. Analyses were performed using S-PLUS statistical software (Version 8.2, TIBCO, Inc., Seattle, WA).

## Results

### GM6001 is Well Tolerated in Naive Dogs

We first addressed the safety of GM6001 using a dose tolerance study. Four healthy dogs were randomized to receive DMSO vehicle, 100 mg/kg GM6001, 150 mg/kg GM6001, or 300 mg/kg GM6001 SC every 12 hours for 3 days. Following drug delivery, all studied parameters were within normal limits, with the following exceptions: 1) increase in body temperature in all GM6001-treated dogs which peaked 6 days after treatment was completed (Fig. S1); 2) transient decrease in food consumption during the 3 days of drug delivery (mean percentage of food consumed, 64.5% ±12.9%) in comparison to the 3 days following delivery (mean percentage of food consumed, 91.7% ±16.3%); and 3) the presence of subcutaneous nodules at the drug delivery sites that regressed in size following delivery in all animals (Fig. S2). No lesions were detected via necropsy or histopathology in the dog that received vehicle. In the dog receiving 300 mg/kg GM6001 twice daily for 3 days, sites of subcutaneous drug deposition were surrounded by a connective tissue capsule with minimal inflammation; additionally, there was mild bile duct hyperplasia. The absence of substantial adverse events in this tolerance study suggested that GM6001 would have an acceptable safety profile in injured dogs.

### GM6001 is Rapidly Detected in Plasma After Subcutaneous Administration

**Figure 1:**
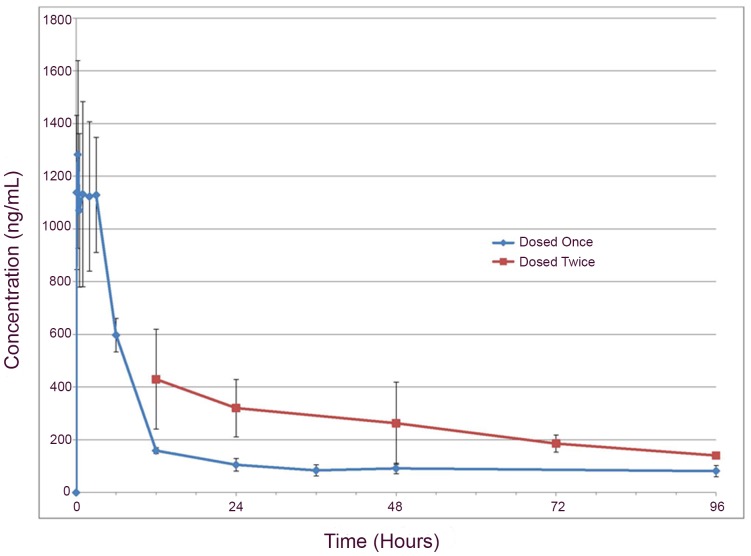
Pharmacokinetics of GM6001 in dogs. Administration of a single 100 mg/kg subcutaneous dose of GM6001 to dogs resulted in the rapid development of peak plasma drug concentrations with drug still detectable 96 hours post-delivery. Administration of a second dose of GM6001 to a sub-group of dogs 12 hours following initial drug delivery resulted in increased plasma drug concentrations at all assessed time points.

**Figure 2:**
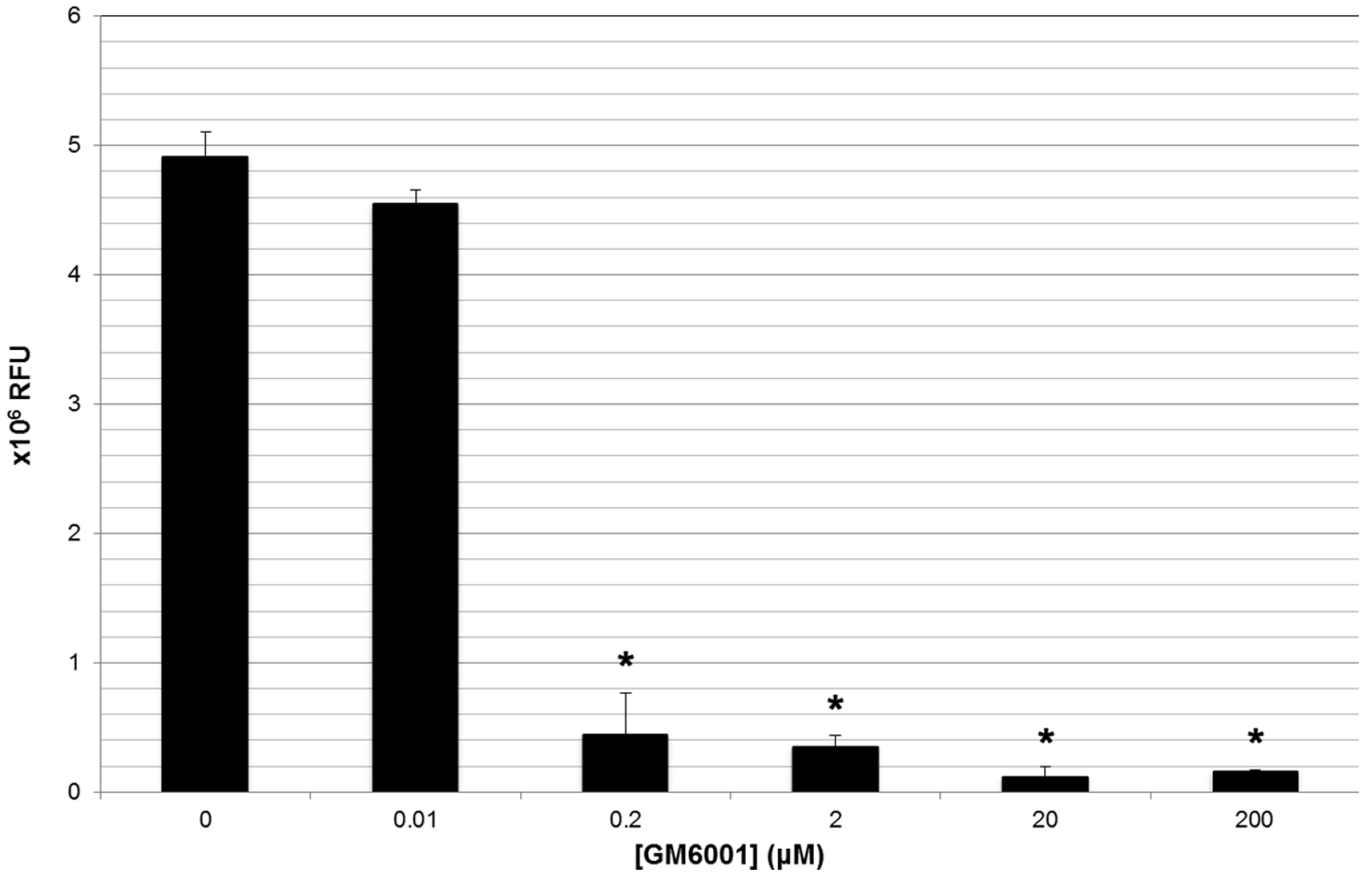
*In vitro* inhibition of MMP-9 by GM6001. Calibrated MMP-9 activity (Log 10^6^), as measured by a fluorescent electrophoretic technique, was dramatically attenuated by GM6001 *in vitro* at various concentrations. Plasma concentrations of GM6001, measured 96 hours following a single 100 mg/kg subcutaenous dose (range 0.16−.26 µM or 60–100 ng/mL), approximated those needed *in vitro* to robustly inhibit MMP-9 (0.2 µM or 77 ng/mL). Groups marked with an asterisk (*) had significantly (P<0.05) different calibrated MMP-9 activity from reference (no GM6001) using a one tailed Student’s t-test.

As the PK of GM6001 might differ from that in rodent [40], we determined the PK in normal dogs. GM6001, administered once at 100 mg/kg SC, was detected in plasma at 5 minutes in all dogs, with a mean time to peak concentration (T_max_) of 0.7 hours (S.D. ±1.3 hours) (Fig. 1). The mean peak concentration (C_max_) was 1370 ng/mL (S.D. ±361 ng/mL), mean apparent elimination half-life (T_1/2_) was 524 hours (S.D. ±428 hours), and the mean plasma concentration of GM6001 at 96 hours was 80 ng/mL (S.D. ±20 ng/mL). Mean area under the curve (AUC) from time 0 to last observed concentration (AUC_0-obs_ ) (16,100 hr*ng/mL ±2981) and mean AUC from time 0 to infinity (AUC_0-∞_ ) (58,225 hr*ng/mL ±37,054) resulted in an extrapolated percentage of AUC of 65%. The only notable adverse event was the presence of focal subcutaneous nodules that regressed with time. The GM6001 utilized in the clinical trial had marked *in vitro* MMP-9 inhibition at concentrations approximating those achieved in dog plasma 96 hours post-drug delivery (Fig. 2). As the objective was to target the acutely injured cord, we selected a single 100 mg/kg SC dose of GM6001 in dogs to achieve plasma drug concentrations which would peak almost immediately after delivery and be sustained at levels sufficient to inhibit MMPs *in vitro* for at least 96 hours following delivery.

### Clinical Trial Enrollment

**Figure 3:**
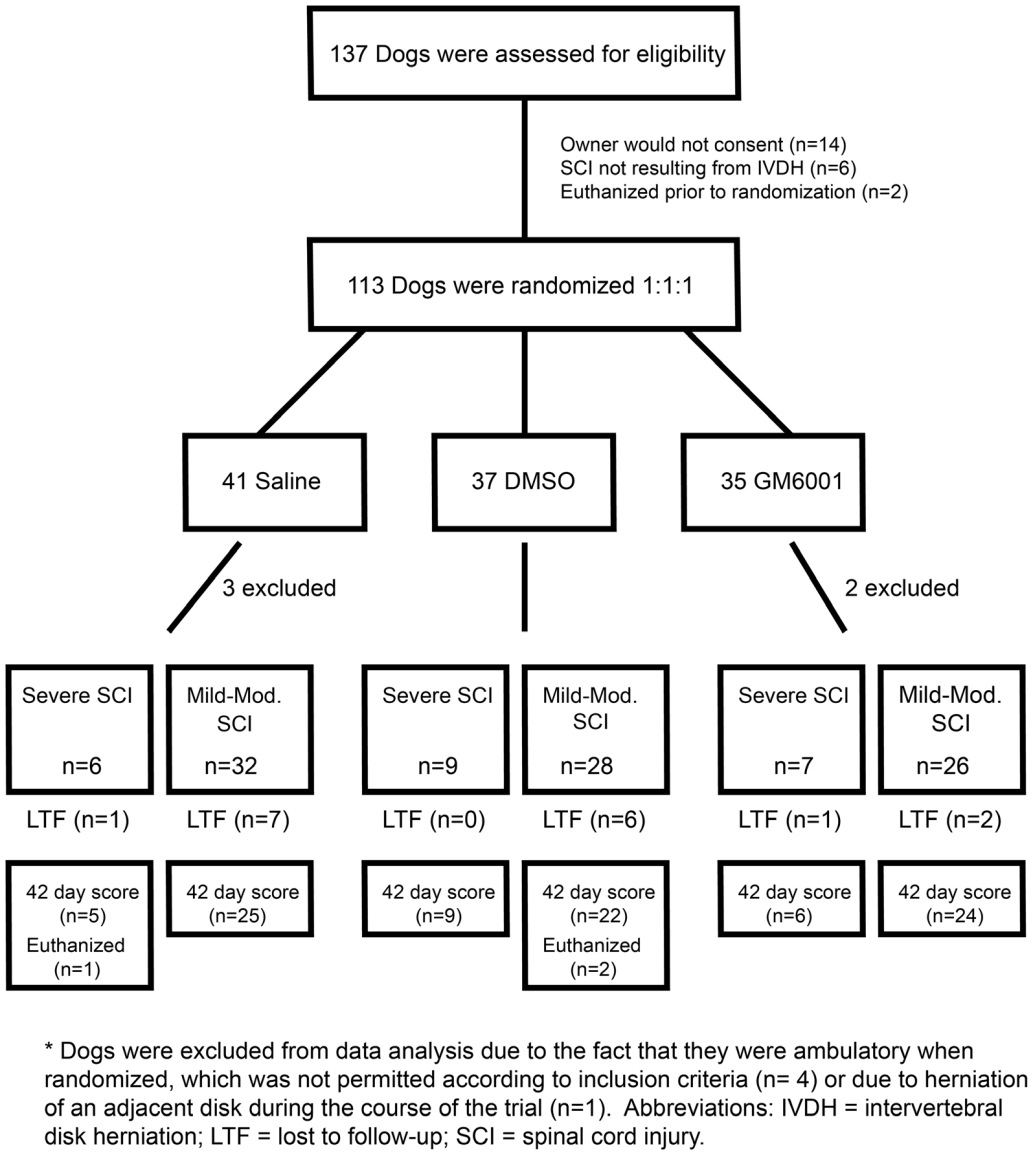
Consolidated Standards of Reporting Trials (CONSORT) Diagram. Flow diagram depicting progress through different phases of the clinical trial including enrollment, group allocation, and follow-up.

Enrolled dogs were randomized to a saline placebo group (n = 38 dogs), a DMSO group (n = 37), and a GM6001 group (n = 33) (Fig. 3). Three dogs were euthanized prior to discharge from the hospital due to neurologic deterioration and 17 dogs did not return for 42-day follow-up examination. Of critical importance, there were no differences in baseline population characteristics such as breed, gender, or injury level among treatment groups, indicating that confounding based on these parameters was unlikely (Table 1).

**Table 1:** Baseline characteristics did not differ significantly among treatment groups.

*A. Continuous variables: Medians (range); P values from Kruskal-Wallis testing*									
Variable		Saline Controls	DMSO			Drug+DMSO		P value	
		*(N = 38)*	*(N = 33)*			*(N = 33)*			
***Age (years)***		5 (2 to 13)	5 (3 to 13)			5 (2 to 14)		0.9833	
***Duration of signs prior to admission (hours)***		24 (1 to 48)	18 (4 to 36)			12 (2 to 48)		0.2246	
MFS^*^		2 (0 to 3)	2 (0 to 3)			2 (0 to 3)		0.7409	
***TSCIS^#^***		4 (0 to 10)	4 (0 to 11)			4 (0 to 10)		0.5907	
***B. Categorical variables: P values from chi-squared testing***									
**Variable**					**DMSO**		**Drug+DMSO**		**P value**
				***(N = 38)***	***(N = 37)***		***(N = 33)***		
***Sex***	**Female**			61% (23/38)	53% (19/36)		39% (13/33)		0.3039
	**Male**			39% (15/38)	47% (17/36)		61% (20/33)		
***Neutered***	**No**			16% (6/38)	22% (8/36)		18% (6/33)		0.9078
	**Yes**			84% (32/38)	78% (28/36)		82% (27/33)		
***Breed***	**Dachshund**			71% (27/35)	61% (22/36)		85% (28/33)		0.1560
	**Other**			29% (8/35)	39% (14/36)		15% (5/33)		
***Chondrodystrophoid***	**Yes**			89% (34/38)	86% (31/36)		88% (29/33)		0.9628
	**Other**			11% (4/38)	14% (5/36)		12% (4/33)		
***Level of Spinal Cord Injury***	**T12-T13**			34% (13/38)	36% (13/36)		24% (8/33)		0.6986
	**T13-L1**			29% (11/38)	25% (9/36)		33% (11/33)		0.8705
	**L1-L2, L2-L3, or L3-L4**			29% (11/38)	33% (12/36)		24% (8/33)		0.8390
***T2-Weighted Hyperintensity (only available for 76 dogs)***	**Absent**			62% (18/29)	76% (22/29)		50% (9/18)		0.3225
	**Present**			38% (11/29)	24% (7/29)		50% (9/18)		

Panel A summarizes continuous variables using medians (ranges) by group, with P values from Kruskal-Wallis tests; panel B describes categorical variables using proportions by group with P values from chi-squared testing. * MFS = Modified Frankel Score; # TSCIS = Texas Spinal Cord Injury Score.

### Adverse Events in Spinal Cord Injured Dogs

Adverse events were recorded during hospitalization and were classified as fever, gastrointestinal, injection site, urinary, or other (Table S1). A significantly greater number of dogs in the GM6001 group had injection site reactions (45%; 15/33) relative to either the saline control dogs (5%; 2/38) or DMSO dogs (0%; 0/36) (P<0.0001; Kruskal-Wallis test). These reactions were transient and consisted of focal dermal and subcutaneous swelling.

### Increased MRI T2W Signal within the Spinal Cord is Associated with Poor Recovery

**Figure 4:**
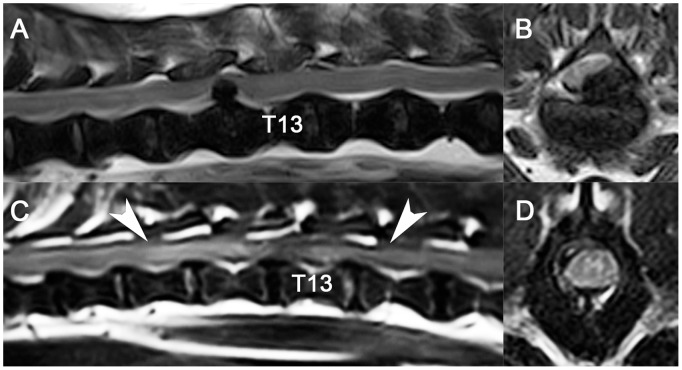
T2-weighted magnetic resonance images in dogs with spinal cord injuries from intervertebral disk herniation. In 1 dog (A, B) that was non-ambulatory with intact pelvic limb movement and sensation, there was focal ventrolateral spinal cord compression at the T12-T13 vertebral articulation without spinal cord signal change. A second dog (C, D) with paraplegia and absent pelvic limb deep nociception had compression at the T12-T13 vertebral articulation. There was extensive spinal cord T2-weighted hyperintensity (white arrows) visible on the sagittal image (C), suggestive of processes seen in contusion injuries such as edema, necrosis, hemorrhage, or cellular infiltrates. The transverse image (D, level of T13 vertebral body) indicated that T2-weighted hyperintensity was predominantly localized to the gray matter.

Vertebral column MRI was performed on 76/107 dogs enrolled in the clinical trial. In all cases, spinal cord compression associated with IVDH was identified with variable presence of increased T2W signal (27/76 dogs) within the spinal cord (Fig. 4). High spinal cord T2 signal, suggestive of contusion, was significantly more common in dogs with severe SCIs (MFS = 0; 11/13) compared with those with mild-to-moderate SCIs (MFS>0; 16/63) (P = 0.0001; Fisher's exact test). Dogs with increased T2W spinal cord also had significantly (P<0.0001; generalized linear model) poorer recovery of function 42 days following SCI (estimated TSCIS 9, 95% CI 7–12) compared to dogs with normal spinal cord T2W signal (estimated TSCIS 15, 95% CI 14–17). The presence of compressive SCI with variable presence of T2W hyperintensity in the spinal cord parallels what is found on MRI in humans with traumatic myelopathy, including relationships between function and spinal cord T2 signal [36].

### Characterization of Cells in CSF Following Spinal Cord Injury

CSF was acquired immediately prior to drug or placebo delivery in 102/107 (95%) clinical trial dogs; all 5 un-injured control animals also had CSF collected. Total nucleated cell count was significantly (P = 0.0034, Wilcoxon rank-sum test) higher in SCI dogs (median = 3 cells/µL, range 0–71) compared with control dogs (median = 0 cells/µL, range 0–1). Amongst dogs with CSF pleocytosis (total nucleated cell count >5 cells/µL), neutrophils were most frequently identified (median 43%, range 2–89%), followed by mononuclear cells (median 25%, range 6–95%) and lymphocytes (median 18%, range 4–70%). CSF red blood cell count was likewise significantly (P = 0.0022, Wilcoxon rank-sum test) increased in dogs with SCI (median 48 cells/µL, range 0–15,040). Together, these findings support a pro-inflammatory state in the acutely injured canine spinal cord.

### GM6001 Reduces Gelatinase Activity in Serum of Spinal Cord Injured Dogs

**Figure 5:**
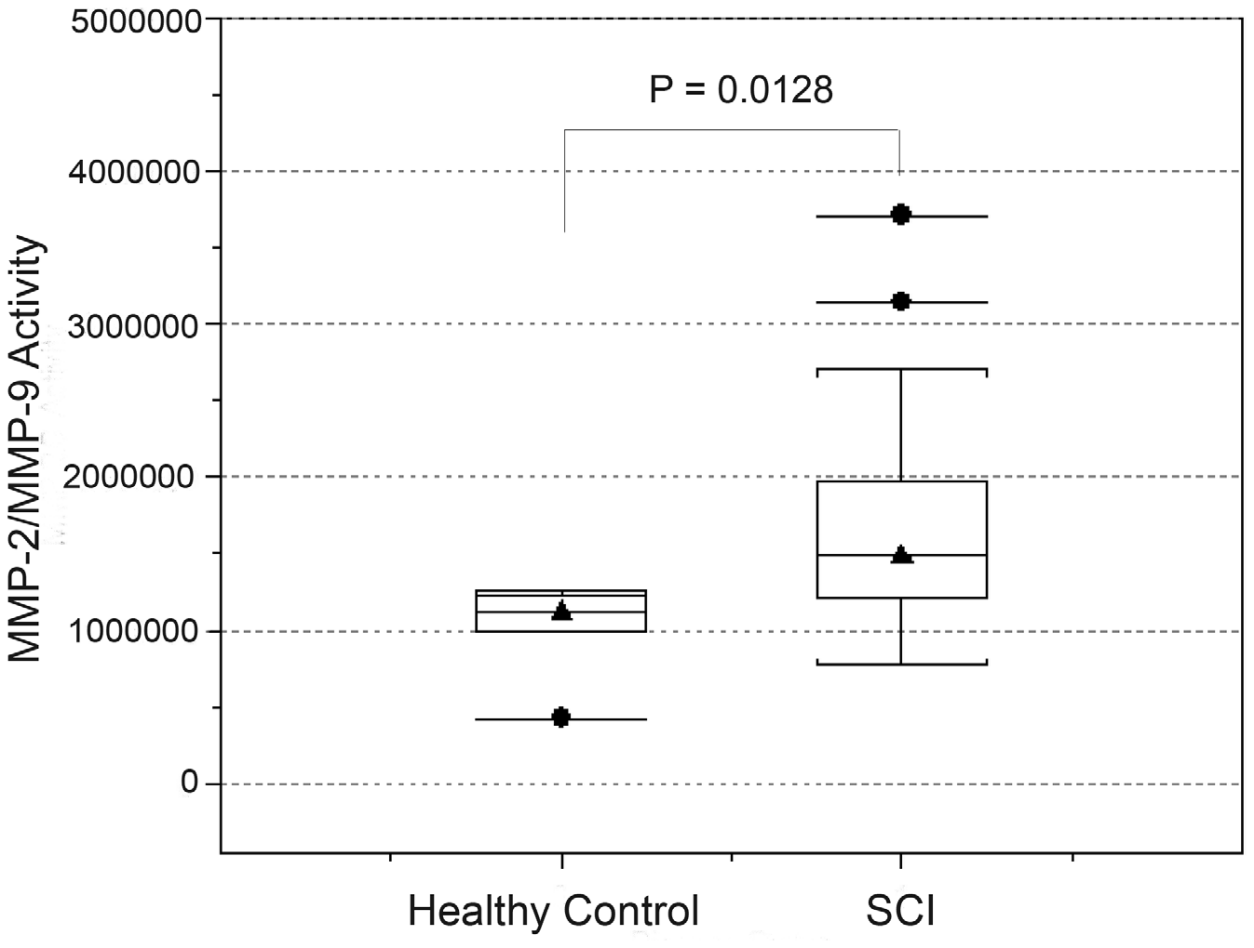
Serum MMP-2/MMP-9 activity in healthy and injured dogs. Box-and-whisker plots summarizing the distribution of MMP 2/9 activity for healthy control dogs (N = 5) and dogs with spinal cord injury (SCI; N = 42) that had serum collected. Values of serum MMP 2/9 activities were significantly (P = 0.0128) greater for dogs with SCI included in the trial than control dogs. The horizontal lines with triangles represent the median value; the horizontal lines at the bottom and top of the boxes represent the 25^th^ and 75^th^ percentiles of the data, respectively. The thin vertical lines extending up or down from the boxes to horizontal lines (so-called whiskers) extend to a multiple of 1.75× the distance of the upper and lower quartile, respectively. Horizontal lines with circles represent values outside the limits of the whiskers.

**Figure 6:**
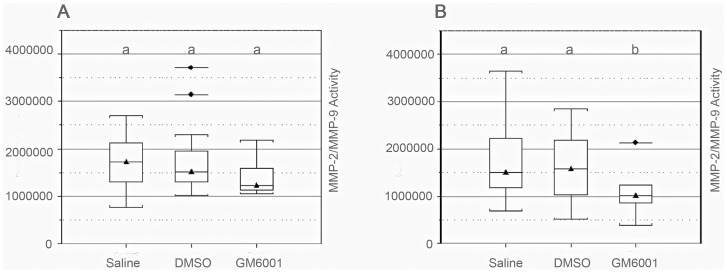
Serum MMP-2/MMP-9 activity pre- and post-drug delivery. Dogs were first randomized into 3 treatment groups, and serum was obtained prior to administration of saline, DMSO, or GM6001 (panel A). There were no differences in serum MMP-2/MMP-9 activity among treatment groups at the time of admission (panel A); however, at day 3, there was a significant (P = 0.0482) difference in serum activity between GM6001-treated dogs and the other treatment groups (panel B). See Figure 5 for a description of box-and-whisker plots. Groups marked with different letters differ significantly (P<0.05).

We utilized a fluorescent electrophoretic technique to determine if MMP-2/MMP-9 activity increases in CSF and serum in dogs with SCI and whether activity is reduced after treatment [37]–[39]. MMP-2/MMP-9 activity in the CSF did not differ between dogs with SCI and control dogs (P = 0.5011; Wilcoxon rank-sum test) (Table S2, Fig. S3). Dogs with SCI had significantly (P = 0.0128; Wilcoxon rank-sum test) higher serum MMP-2/MMP-9 activity prior to treatment compared with control dogs, but activity did not vary based on clinical factors or MRI features of SCI (Table 2, Fig. 5). Serum MMP-2/MMP-9 activity was significantly (P<0.05; generalized linear model) lower in dogs receiving GM6001 3 days following treatment compared to dogs receiving either DSMO or saline (Fig. 6). Thus, these findings confirm the effectiveness of GM6001 in reducing the abnormal elevation of MMP-2/MMP-9 in serum of spinal cord injured dogs.

**Table 2:** Values of serum MMP-2/MMP-9 activity were not significantly associated with various clinical variables.

Variable	Median (Range) of MMP-2/MMP-9 Activity in Serum		P value
***Age***	***≤5 Years (N = 28)***	***>5 Years (N = 12)***	
	1,519,998 (1,009,205–3,708,449)	1,315,180 (769,191–2,700,981	0.1458
***Sex***	***Male (N = 22)***	***Female (N = 18)***	
	1,504,968 (1,009,205–3,708,449)	1,455,587 (769,191–2,700,981)	0.2625
***Neutered***	***Not neutered (N = 9)***	***Neutered (N = 31)***	
	1,492,947 (1,009,205–2,266,638)	1,479,017 (769,191–3,708,449)	0.5881
***Breed***	***Dachshund (N = 28)***	***Other (N = 12)***	
	1,411,546 (769,191 - 3,144,395)	1,528,755 (941,766 - 3,708,449)	0.4932
***Chondrodysplastic***	***Yes (N = 38)***	***Other (N = 2)***	
	1,474,618 (769,191–3,708,449)	1,983,362 (1,975,379–1,991,345)	0.2564
***Duration of clinical signs prior to admission***	***≤12 hours (N = 12)***	***>12 hours (N = 28)***	
	1,453,961 (1,009,205 - 3,708,449)	1,485,982 (769,191–3,144,395)	0.9189
	***≤24 hours (N = 35)***	***>24 hours (N = 5)***	
	1,492,947 (941,766–3,708,449)	1,310,144 (769,191–2,306,259)	0.5243
***T2-weighted hyperintensity***	***Absent (N = 20)***	***Present (N = 11)***	
	1,507,977 (769,191–3,708,449)	1,523,790 (1,062,500–3,144,395)	0.6995
***MFS at admission***	***≤2 (N = 22)***	***>2 (N = 18)***	
	1,455,587 (1,009,205 - 2,292,539)	1,504,968 (769,191–3,708,449)	0.4924

Medians (and ranges) and P values derived from Wilcoxon-rank sum tests are reported for the categorical variables listed above. MMP = matrix metalloproteinase; MFS = Modified Frankel Score.

### DMSO Enhances Recovery in Dogs with Severe Spinal Cord Injuries

**Figure 7:**
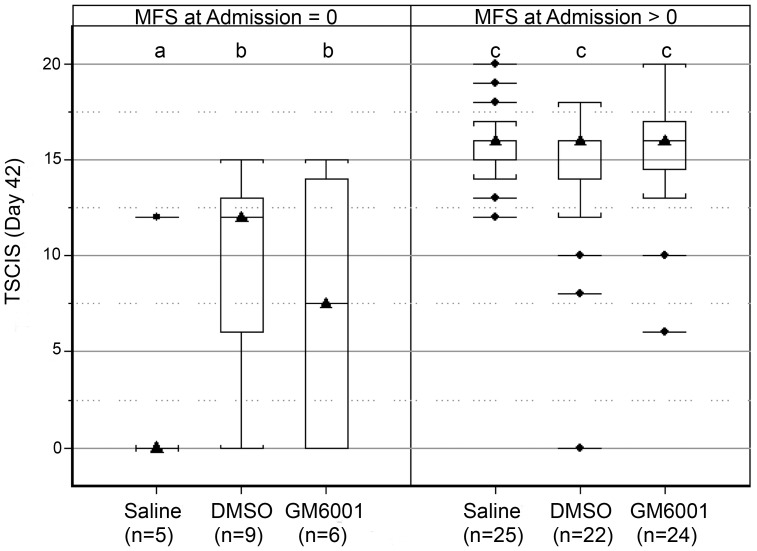
Evaluation of primary outcome in dogs with SCI. Box-and-whisker plots of TSCIS on day 42 by treatment group, stratified by MFS at admission (MFS = 0, left panel; or MFS >0, right panel). There were no significant differences in TSCIS among dogs with MFS score >0 (right panel), but TSCIS was significantly (P<0.05) greater for the GM6001 and the DMSO group than saline treated dogs with MFS = 0 (left panel). See Figure 5 for a description of box-and-whisker plots. Groups marked with different letters differ significantly (P<0.05).

In dogs with mild-to-moderate SCI (i.e., MFS >0), there was robust recovery of function by 42 days with 64 of 71 (90%) dogs independently walking and 69 of 71 (97%) having intact pelvic limb nociception. Treatment group did not influence 3 or 42 day TSCIS (Fig. 7, Fig. S4). Dogs with mild-to-moderate SCI had significantly higher 42-day TSCIS (mean 15; 95% CI 12–18) compared to those with severe SCI (mean 7; 95% CI 4–9) (P<0.0010; generalized linear model).

**Figure 8:**
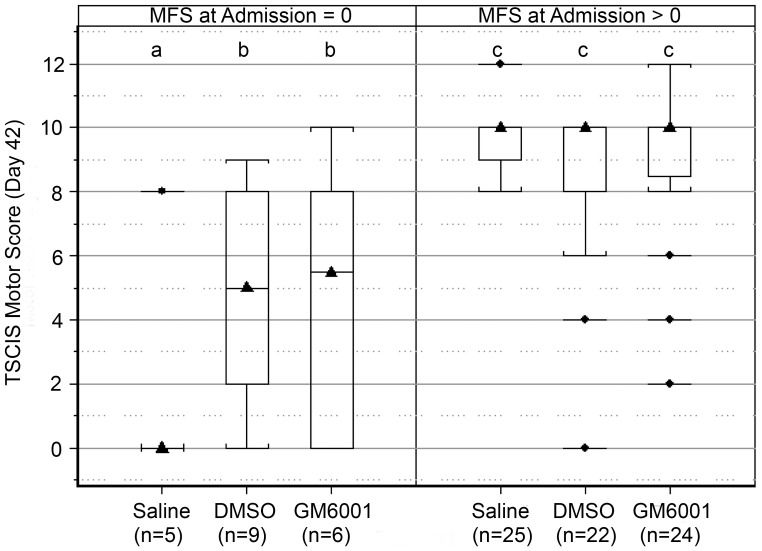
Evaluation of TSCIS motor score at day 42 following SCI. Box-and-whisker plots of TSCIS motor score on day 42 by treatment group, stratified by MFS at admission (MFS = 0, left panel; or MFS >0, right panel). There were no significant differences in motor score among dogs with MFS >0 (right panel), but motor score was significantly (P<0.05) greater for the GM6001 and the DMSO group than saline treated dogs with MFS = 0 (left panel). See Figure 5 for a description of box-and-whisker plots. Groups marked with different letters differ significantly (P<0.05).

In dogs with severe SCI (i.e., MFS = 0), those receiving either DMSO or GM6001 had significantly (P<0.05; generalized linear model) more robust functional recovery compared with those receiving saline placebo (Fig. 7) at 42 days. Sub-components of the 42-day TSCIS were examined in dogs with severe SCI to better capture the influence of treatment on motor, sensory, and postural recovery. Sensory and postural scores did not differ significantly between treatment groups. Dogs receiving saline had an estimated mean motor score of 2 (95% CI 0–4.0) (suggesting absent to minimal pelvic limb movement with tail support) that was significantly (P<0.05; generalized linear model) less than the estimated mean motor score for dogs receiving DMSO (mean, 5; 95% CI 2.0–8.0) or GM6001 (mean, 5; 95% CI 2.0–8.0) (Fig. 8). The distribution of motor scores for both the DMSO and GM6001 treated dogs indicated that the majority of animals in these groups developed coordinated stepping movements with tail support and of those regaining movement many (6 of 12; 50%) walked without any support. Dogs that were treated with DMSO or GM6001 that regained pelvic limb movement typically (10 of 12; 83%) also recovered limb nociception. The extent of neurological recovery at 42 days post-injury was not significantly different in DMSO- and GM6001 (dissolved in DMSO)-treated groups. Such findings suggest that DMSO, rather than GM6001 contributed to enhanced recovery in these treatment groups.

## Discussion

This study was designed as a large-scale clinical trial to evaluate MMP inhibition in a clinically relevant, naturally occurring canine SCI model. Using advanced technology to measure activity of MMP-2/MMP-9, we show that these proteases are elevated in serum of dogs across all levels of injury severity and that GM001, given as a single bolus subcutaneously, significantly reduced this activity. Despite the effectiveness of GM6001 in targeting early MMP activity, both GM6001, solubilized in DMSO, and DMSO alone produced similar levels of neurological improvement in dogs with severe SCIs, relative to saline controls. At 42 days post-injury, these dogs showed robust stepping movements that were visible with tail support and many independently ambulated; saline-treated dogs either showed no movement or had minimal limb advancement without stepping. Together, these findings demonstrate that early blockade of MMPs did not improve long-term neurological recovery. Rather, DMSO alone was responsible for the beneficial outcomes in dogs with severe SCIs.

The clinical trial described here was designed to include dogs with both severe (paraplegia and absent nociception) and mild-to-moderate SCIs (non-ambulatory with intact nociception) for several reasons. First, there is an abnormal elevation of MMP-9 in serum [20], CSF [20], [41] and spinal cords of dogs [42] with IVDH across a spectrum of injury severities. Second, while long-term recovery of ambulation is common (64 of 71 dogs in this trial walked independently) in the mild-to-moderate injury group, few animals normalize with reference to motor or postural scores [43]. Thus, there is an opportunity, even within animals that are likely to show marked recovery, to examine the effect of therapeutics. We chose to stratify our population based on SCI severity to examine the effect of treatment on neurologic recovery. This approach was necessary given the well-known difference in outcome between these populations (approximately 85–95% of mildly-to-moderately and 50–60% of severely injured dogs recover independent ambulation) and the potential for differential activation of secondary injury pathways based on SCI severity [30]–[33]. Stratification based on SCI severity is common and accepted in human clinical trials because of expected differences in recovery between injury groups and the potential impact of this difference on evaluation of effectiveness of therapies [44], [45].

GM6001 is a broad-spectrum MMP inhibitor that has been shown to exert neuroprotection in rodent models of brain and SCIs, primarily via antagonism of MMP-9 associated with neutrophils [1]. Evidence supporting this position includes a temporal association between neutrophil trafficking and MMP-9 expression, reduced expression of MMP-9 in spinal cord injured mice that are neutrophil-depleted, and reduced neutrophil content within injured spinal cords of MMP-9 null mice [2], [8], [46]. In this study, GM6001 was delivered SC using DMSO as a vehicle. While the high prevalence of injection site reactions and route of administration may have altered drug absorption in comparison to studies in other species, our data support favorable PK via SC administration of GM6001. Furthermore, relatively small plasma concentrations of GM6001 present 3 days post-delivery appear capable of modulating MMP-2/MMP-9 activity in study dogs.

Here we studied the effects of GM6001 on MMP-2/MMP-9 activity in serum. While there was no relationship between injury severity and level of MMP-2/9 activity in serum, spinal cord injured dogs showed an increase in these proteases relative to healthy controls. Moreover, the early elevation of serum MMP-2/MMP-9 activity was significantly reduced following treatment with GM6001, a finding which serves to confirm the effectiveness of the drug in reducing proteolytic activity.

While MMP-2/MMP-9 activity was detected in the CSF of injured dogs, activity did not differ between healthy control dogs and those with SCI. The lack of a demonstrable difference in CSF MMP-2/MMP-9 between SCI and control groups may reflect the inability of this assay to distinguish between the 2 proteases. Based upon an earlier study using gelatin zymography [20], MMP-2 was found to be expressed in the CSF of normal dogs and remained unchanged after SCI. In contrast, MMP-9 was only detected in spinal cord injured dogs [20], [41]. Thus, in the current study, the absence of any differences between injured and control dogs may have been confounded by the constitutive activity of MMP-2 in CSF that may have masked any increase in MMP-9.

There are likely a number of possible explanations for why GM6001 failed to improve neurological recovery in spinal cord injured dogs. First, while GM6001 has been shown to improve neurological outcomes in various rodent models of brain and spinal cord injury [2], [47], [48], no studies to date have evaluated efficacy in dogs. Thus, there may be species differences in responsiveness to GM6001 and/or MMP-directed pathogenesis. Additionally, effects of GM6001 demonstrated in rodents may not be sufficiently robust to positively influence outcome under the clinical conditions of this study [49], [50]. Second, the drug was active beyond the first several days post-injury and as such could have interfered with mechanisms underlying recovery in SCI. Pharmacokinetics in healthy dogs demonstrated that plasma concentration of GM6001, present at even the 96-hour time-point, approximated or exceeded that necessary to block MMP-9 *in vitro*. As some MMPs modulate the formation of a glial scar and axonal plasticity [4], their subacute/chronic blockade may result in adverse neurological outcomes. Third, the timing between SCI and administration of GM6001 may not have been optimal. The strong association between MMP-9 expression and neutrophils suggests that an optimal therapeutic window for GM6001 is defined by the early trafficking of neutrophils into the injured cord. Such a position is supported by evidence of pronounced neurological recovery when the drug was given beginning 3 hours post-injury in a murine model of SCI [2]. In dogs treated with GM6001, median delay between injury and enrollment was 12 hours, which may have exceeded the window of efficacy for GM6001. Finally, while the use of dogs with thoracic and lumbar spinal cord lesions could have influenced our ability to detect drug-related effects [51], the proportion of dogs with lumbar lesions was similar amongst treatment groups (Table 1). Additionally, the inclusion of lesion location (lumbar versus thoracic) in multivariable generalized linear modeling did not alter the significance or magnitude of observed treatment effects (data not shown).

We found that DMSO improved motor recovery in dogs with severe SCIs. This finding is perhaps not too surprising as DMSO, under defined dosing conditions, has the ability to function as a neuroprotectant [14] and in some cases when used as a vehicle, may be synergistic. In the setting of neurotrauma, neuroprotection is exemplified in a study by Di Giorgio et al [52] which compared the antioxidant curcumin, α-tocopherol, DMSO and saline in a model of traumatic brain injury. These authors reported similar levels of early neuroprotection across all agents relative to the saline control group. Beneficial effects of DMSO might also be indicated in studies where DMSO is used as a vehicle without any additional negative control group. For example, in a recent study the efficacy of an epidermal growth factor receptor inhibitor was assessed in a rodent model of SCI [53]. This inhibitor was compared against its vehicle, DMSO. Recovery of motor and bladder function was significantly greater in rats that received DMSO relative to the inhibitor. The authors concluded that the receptor inhibitor showed no efficacy relative to the “baseline” values as defined by DMSO. Based upon our study, an alternative explanation is that DMSO did not serve as the “baseline” but rather may have exerted a beneficial effect. Finally, DMSO, when co-administered with a candidate therapeutic, offers potential for synergism, by acting through separate and/or overlapping pathways. While we found no evidence of this in the current study, others have reported synergism in a model of brain ischemia where DMSO was either combined with fructose 1,6-disphosphate, an intermediate of anaerobic metabolism, or prostacyclin (PG_I2_) which blocks aggregation of platelets and functions as a vasodilator [12], [54].

The mechanisms underlying DMSO-mediated neuroprotection have been attributed to its ability to function as a free radical scavenger and suppress a variety of pathobiologic events including inflammation, calcium influx, and glutamate excitoxicity [14]. Such broad-based, temporally-defined targets may account for the extended window of efficacy (within 48 hours of injury) in spinal cord-injured dogs.

Dogs with severe SCIs and treated with either DMSO or GM6001 in DMSO showed a consistent (>80%), improvement in pelvic limb stepping. A critical question is whether this stepping was voluntary or mediated through the central pattern generator (i.e., spinal stepping). The vast majority of these dogs with motor recovery also regained pelvic limb nociception (10 of 12; 83%) and 50% walked independently (without tail support) when evaluated at 42 days post injury. These data would argue that pelvic limb movement was indeed voluntary in the majority of dogs with severe SCIs treated with either DMSO or GM6001.

In summary, while this study and others [12], [13], [52], [54] underscore the potential utility of DMSO for the treatment of brain and SCI, there remain conflicting reports about the efficacy of DMSO. This is illustrated in recent studies reporting either no effects or reduced performance on behavioral tests after traumatic brain injury [55] and others suggesting improved learning ability in cerebellar mutant Lurcher mice [56]. As it is shrouded in controversy as a therapeutic yet commonly used as a vehicle, there is a need to rigorously evaluate DMSO from the standpoint of safety, dosing, and efficacy. Given that it is a common vehicle for drug delivery, there is opportunity to evaluate its synergistic properties. Such logic has been successfully applied to the treatment of human interstitial cystitis, where DMSO is given as part of multimodal regimen [57]. With FDA approval for the treatment of interstitial cystitis, there is potential for the repurposing of DMSO, capitalizing on its favorable properties as a solvent, in developing combinatorial therapies for SCI. The current study suggests that DMSO has an extended therapeutic window (up to 48 hours). As time to treatment for human SCI may be delayed for up to 1 to 3 days post-injury [58], a broader therapeutic window could potentially expand the population of spinal cord injured patients that would otherwise not qualify for treatments with more restricted windows of intervention.

## Supporting Information

Figure S1 Rectal body temperature in healthy dogs delivered DMSO or GM6001. All dogs delivered GM6001 at 6–18 times the cumulative clinical trial dose (100–300 mg/kg six times) experienced body temperature elevations beyond normal. The elevation in body temperature qualitatively appeared greatest in dogs receiving higher doses of GM6001.
(TIF)

Figure S2 Drug delivery site diameters in healthy dogs receiving GM6001. Delivery site diameter appeared greatest one day after administration (panel A) and diminished by day 8 post-administration (panel B) in dogs receiving 6–18 times the cumulative clinical trial dose of GM6001.
(TIF)

Figure S3 Cerebrospinal fluid MMP-2/MMP-9 activity in healthy and spinal cord injured dogs. Although MMP-2/MMP-9 activity tended to be higher in dogs with spinal cord injuries (n = 40) than healthy controls (n = 5), the difference was not significant (P = 0.5011; Wilcoxon rank-sum test).
(TIF)

Figure S4 Texas Spinal Cord Injury Score (TSCIS) on day 3 following spinal cord injury. There were no significant differences in TSCIS based on treatment group for dogs with severe (MFS = 0) and mild-to-moderate (MFS >0) spinal cord injuries. Box-and-Whiskers with different letters differ significantly (P<0.05).
(TIF)

Table S1 Frequency of adverse events and survival (died or euthanized) by treatment group. Injection site reactions were significantly associated with GM6001 delivery, but no other adverse events were significantly associated with treatments.
(DOCX)

Table S2 Cerebrospinal MMP-2/MMP-9 activity in dogs with spinal cord injury. Cerebrospinal fluid MMP-2/MMP-9 activity in dogs with spinal cord injury was not significantly associated with signalment, duration of clinical signs, or MFS at the time of admission. Medians (and ranges) and P values derived from Wilcoxon-rank sum tests were reported for the categorical variables.
(DOCX)
