# Supplementary figures and images for: Efficacy of a Metalloproteinase Inhibitor in Spinal Cord Injured Dogs
Source: PLoS One. 2014 May 1;9(5):e96408. doi: 10.1371/journal.pone.0096408 (PMC4006832; doi:10.1371/journal.pone.0096408)

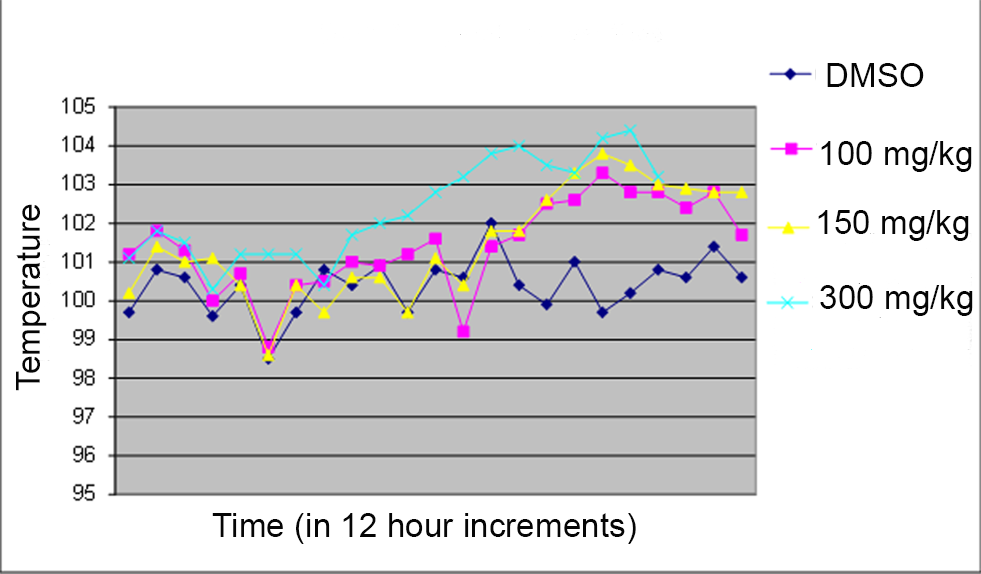

Supplement: Figure S1 — Rectal body temperature in healthy dogs delivered DMSO or GM6001. All dogs delivered GM6001 at 6–18 times the cumulative clinical trial dose (100–300 mg/kg six times) experienced body temperature elevations beyond normal. The elevation in body temperature qualitatively appeared greatest in dogs receiving higher doses of GM6001. (TIF) [file pone.0096408.s001.tif]

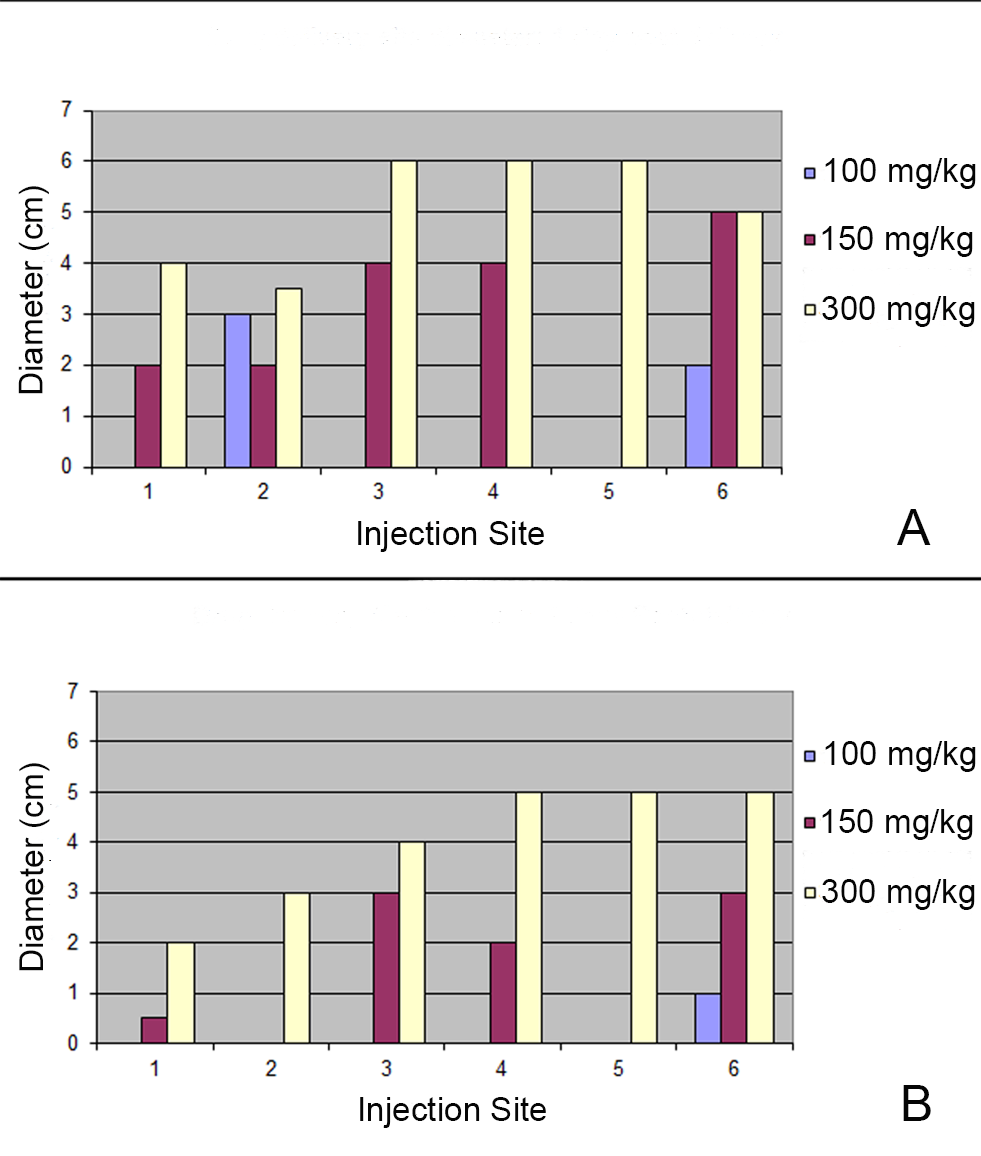

Supplement: Figure S2 — Drug delivery site diameters in healthy dogs receiving GM6001. Delivery site diameter appeared greatest one day after administration (panel A) and diminished by day 8 post-administration (panel B) in dogs receiving 6–18 times the cumulative clinical trial dose of GM6001. (TIF) [file pone.0096408.s002.tif]

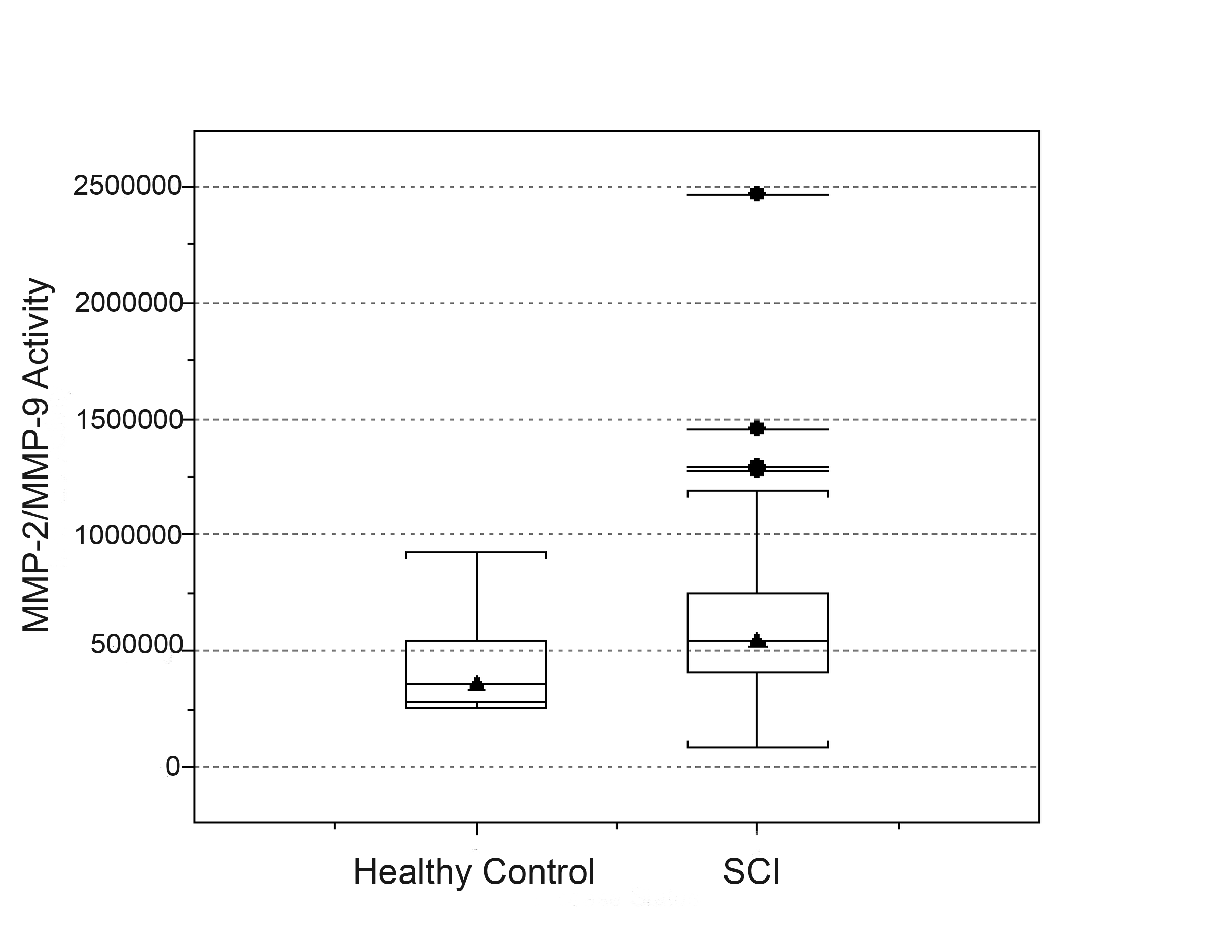

Supplement: Figure S3 — Cerebrospinal fluid MMP-2/MMP-9 activity in healthy and spinal cord injured dogs. Although MMP-2/MMP-9 activity tended to be higher in dogs with spinal cord injuries (n = 40) than healthy controls (n = 5), the difference was not significant (P = 0.5011; Wilcoxon rank-sum test). (TIF) [file pone.0096408.s003.tif]

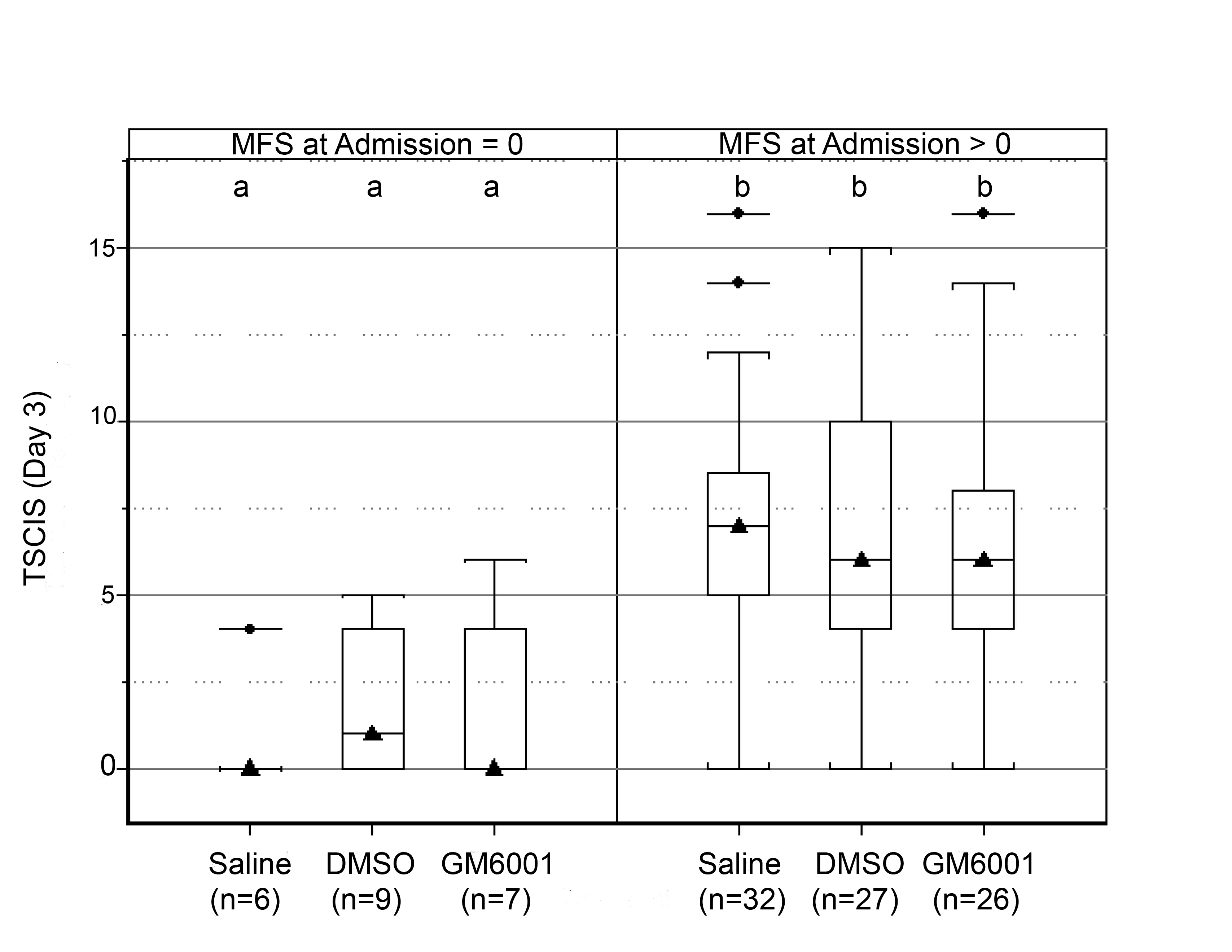

Supplement: Figure S4 — Texas Spinal Cord Injury Score (TSCIS) on day 3 following spinal cord injury. There were no significant differences in TSCIS based on treatment group for dogs with severe (MFS = 0) and mild-to-moderate (MFS >0) spinal cord injuries. Box-and-Whiskers with different letters differ significantly (P<0.05). (TIF) [file pone.0096408.s004.tif]
