# Supplementary material for: Efficacy of a Metalloproteinase Inhibitor in Spinal Cord Injured Dogs
Source: PLoS One. 2014 May 1;9(5):e96408. doi: 10.1371/journal.pone.0096408 (PMC4006832; doi:10.1371/journal.pone.0096408)
Supplement: Table S1 — Frequency of adverse events and survival (died or euthanized) by treatment group. Injection site reactions were significantly associated with GM6001 delivery, but no other adverse events were significantly associated with treatments. (DOCX) [file pone.0096408.s005.docx]

Supplemental Table 1: **Frequency of adverse events and survival (died or euthanized) by treatment group.**

| **Adverse Event** | **Saline Control** | **DMSO** | **Drug** | **P value** |
| --- | --- | --- | --- | --- |
| **Any** | 29% (11/38) | 28% (10/36) | 55% (18/33) | 0.0682 |
|  |  |  |  |  |
| **Injection site reaction** | 5% (2/38) | 0% (0/36) | 45% (15/33) | < 0.0001 |
|  |  |  |  |  |
| **Gastrointestinal problems** | 16% (6/38) | 25% (9/36) | 24% (8/33) | 0.6097 |
| **Anorexia** | 0% (0/38) | 3% (1/36) | 0% (0/33) | ND |
| **Diarrhea** | 10% (4/38) | 8% (3/36) | 15% (5/33) | ND |
| **Regurgitation** | 0% (0/38) | 3% (1/36) | 0% (0/33) | ND |
| **Vomiting** | 3% (1/38) | 11% (4/36) | 3% (3/33) | ND |
| **Vomiting + diarrhea** | 3% (1/38) | 0% (0/36) | 0% (0/33) | ND |
|  |  |  |  |  |
| **Urinary tract infection** | 5% (2/38) | 3% (1/36) | 3% (1/33) | 1.0000 |
|  |  |  |  |  |
| **Fever** | 3% (1/38) | 0% (0/36) | 3% (1/33) | 0.7588 |
|  |  |  |  |  |
| **Other adverse events** | 3% (1/38) | 6% (2/36) | 0% (0/33) | 0.6451 |
| **Seizures** | 3% (1/38) | 0% (0/36) | 0% (0/33) | ND |
| **Pneumonia** | 0% (0/38) | 3% (1/36) | 0% (0/33) | ND |
| **Cardiovascular** | 0% (0/38) | 3% (1/36) | 0% (0/33) | ND |
|  |  |  |  |  |
| **Died or euthanized** | 3% (1/38) | 6% (2/36) | 0% (0/33) | 0.6451 |

Injection site reactions were significantly associated with the study treatment, but no other adverse events were significantly associated with treatment. ND= not determined.
