# Supplementary material for: Efficacy of a Metalloproteinase Inhibitor in Spinal Cord Injured Dogs
Source: PLoS One. 2014 May 1;9(5):e96408. doi: 10.1371/journal.pone.0096408 (PMC4006832; doi:10.1371/journal.pone.0096408)
Supplement: Table S2 — Cerebrospinal MMP-2/MMP-9 activity in dogs with spinal cord injury. Cerebrospinal fluid MMP-2/MMP-9 activity in dogs with spinal cord injury was not significantly associated with signalment, duration of clinical signs, or MFS at the time of admission. Medians (and ranges) and P values derived from Wilcoxon-rank sum tests were reported for the categorical variables. (DOCX) [file pone.0096408.s006.docx]

Supplemental Table 2: **Cerebrospinal MMP-2/MMP-9 activity in dogs with spinal cord injury**.

| **Variable** | **Median (Range) of MMP 2/9 Activity in CSF** | | **P value** |
| --- | --- | --- | --- |
| ***Age*** | ***< 5 Years (N = 29)*** | ***> 5 Years (N =13)*** |  |
|  | 528,300 (90,722 - 2,465,990) | 623,624 (90,576 – 1,192,415) | 0.8596 |
| ***Sex*** | ***Male (N = 22)*** | ***Female (N = 20)*** |  |
|  | 562,403 (90,576 – 2,465,990) | 513,502 (90,722 - 1,192,415) | 0.3449 |
| ***Neutered*** | ***Not neutered (N = 9)*** | ***Neutered (N = 33)*** |  |
|  | 494,948 (94,915 – 1,455,369) | 555,397 (90,576 - 2,465,990) | 0.7130 |
| ***Breed*** | ***Dachshund (N = 28)*** | ***Other (N = 14)*** |  |
|  | 526,151 (90,576 – 1,455,369) | 637,540 (94,915 - 2,465,990) | 0.3106 |
| ***Chondrodysplastic*** | ***Yes (N=39)*** | ***Other (N=3)*** |  |
|  | 528,300 (90,576 – 1,455,369) | 591,240 (503,002 - 2,465,990) | 0.3051 |
| ***Duration of clinical signs prior to admission*** | ***< 12 hours (N = 12)*** | ***> 12 hours (N = 30)*** |  |
|  | 524,524 (90,722 - 2,465,990) | 541,849 (90,576 – 1,275,724) | 0.8129 |
|  | ***< 24 hours (N = 37)*** | ***> 24 hours (N = 5)*** |  |
|  | 528,300 (90,576 – 2,465,990) | 751,791 (207,229 - 775,929) | 0.6551 |
| ***T2-weighted hyperintensity*** | ***Absent (N = 21)*** | ***Present (N = 11)*** |  |
|  | 555,397 (94,915 – 2,465,990) | 629,071 (90,722 – 1,455,369) | 0.7504 |
| ***MFS at admission*** | ***< 2 (N = 23)*** | ***> 2 (N = 19)*** |  |
|  | 528,300 (90,576 – 2,465,990) | 555,397 (94,915 – 1,290,030) | 0.8795 |

Cerebrospinal fluid MMP-2/MMP-9 activity in dogs with spinal cord injury was not significantly associated with signalment, duration of clinical signs, or MFS at the time of admission. Medians (and ranges) and P values derived from Wilcoxon-rank sum tests were reported for the categorical variables listed above. MFS = Modified Frankel Score
